# The impact of online real-time teaching interaction on teaching effect among undergraduate nursing students: the mediating role of deep learning

**DOI:** 10.3389/fpubh.2026.1715936

**Published:** 2026-01-30

**Authors:** Dongmei Liu, Wanpeng Zhen, Shenzhen Yi, Yongchao Jin, Ye Lin, Rui Yang

**Affiliations:** 1 School of Science, North China University of Science and Technology, Tangshan, China; 2 School of Psychology and Mental Health, North China University of Science and Technology, Tangshan, China; 3 School of Philosophy and Sociology, Jilin University, Changchun, China

**Keywords:** deep learning, mediating role, online real-time teaching interaction, teaching effect, undergraduate nursing students

## Abstract

**Background:**

With the deep integration of information technology and education, online teaching has become an important form of nursing education. However, students may encounter problems such as low participation and difficulty in internalizing knowledge during online learning. Existing studies have shown that deep learning plays a key role in promoting students’ learning outcomes and professional skills development, but its mediating role between online real-time teaching interaction and teaching effect among undergraduate nursing students has not been fully studied.

**Objective:**

This study aims to explore the relationships among online real-time teaching interactions, deep learning, and online teaching effect for undergraduate nursing students, with a focus on analyzing the mediating role of deep learning in the relationship between online real-time teaching interactions and teaching effect.

**Methods:**

A total of 587 nursing students from the universities in Hebei Province offering nursing programs were recruited via convenience sampling to complete the questionnaire survey. The research instruments included the online real-time teaching interaction scale, the deep learning scale, and the teaching effect scale. Data analysis was performed using SPSSAU, which involved descriptive statistics, correlation analysis, regression analysis, and a test of the mediating effect of deep learning.

**Results:**

There is a significant positive correlation between deep learning and teaching effect among undergraduate nursing students (*r* = 0.407, *p* < 0.01), as well as between online real-time teaching interactions and teaching effect (*r* = 0.398, *p* < 0.01). Deep learning acts as a partial mediator in the relationship between online real-time teaching interactions and teaching effect, with a mediating effect value of 0.165, accounting for 36.344% of the total effect.

**Conclusion:**

This study reveals that online real-time teaching interaction not only directly and positively predicts the teaching effect of undergraduate nursing students but also exerts an indirect influence by enhancing their deep learning levels. The results indicate that in online nursing education, the intentional design of interactive segments that promote deep learning is crucial. Educators should focus on constructing an interactive environment capable of stimulating higher-order thinking among nursing students to more effectively enhance the quality of online teaching.

## 1 Introduction

Improving teaching quality is the core task of higher education and also a basic requirement for building an educational power (1, 2). The rapid evolution of information technology and its integration into pedagogical practices have brought about significant transformations in the global higher education landscape (3, 4). This shift is especially consequential in fields that necessitate hands-on training, such as nursing education (5). The COVID-19 pandemic acted as a transformative force, precipitating an abrupt transition to remote learning, a model that has since developed into a sustained reliance on both hybrid and fully online modalities (6, 7). Despite its advantages in flexibility and accessibility, the effectiveness of online modalities in fostering the sophisticated skills essential for future healthcare professionals continues to be a pivotal research question. Nursing education extends beyond the acquisition of foundational knowledge, requiring the development of advanced competencies, including clinical reasoning, critical thinking, empathy, and collaborative skills. These essential capabilities have traditionally been nurtured through dynamic interpersonal interactions in both clinical environments and classroom settings (8, 9).

Interaction is defined as the process of reciprocal actions or responses occurring between individuals or groups (10). Within an educational context, instructional interaction encompasses the verbal and non-verbal communication between instructors and students, as well as among learners themselves (11). Synchronous online instruction leverages a variety of digital tools to broaden the scope of these interactions and enhance pedagogical efficacy (12, 13). Real-time interaction, a cornerstone of this modality, enables bidirectional communication between instructors and students at the same moment. This simultaneity profoundly influences key educational dynamics: it shapes the classroom atmosphere (14), affects learner engagement and motivation (15), and facilitates the construction of connections between new and prior knowledge (16). Consequently, the quality of real-time interaction is a critical determinant in achieving desired learning outcomes and instructional objectives. As a modern pedagogical approach, synchronous online interaction has emerged as a significant pathway for improving teaching effect. Its primary advantage lies in providing a more flexible and diverse learning environment. Students can tailor their learning schedules and methods according to their individual progress and comprehension. Through real-time questioning, discussion, and feedback, they can promptly resolve difficulties, thereby deepening their understanding and mastery of the subject matter (17). Concurrently, instructors can utilize online tools and learning analytics to monitor student progress more effectively, assess instructional impact, and adapt teaching content and strategies based on student feedback and data, enabling a more precise and personalized teaching approach. This highly interactive mode of instruction not only increases student motivation and self-directed learning but also fosters the development of critical thinking and innovative capacities.

Distinct from “machine deep learning” in the field of computer science, “human deep learning” in education focuses on the deep engagement and advanced development of learners’ cognition, emotion, and behavior. Its core lies in the internalization of knowledge, enhancement of abilities, and formation of competencies through active construction, meaning integration, and transferable application, rather than passive memorization or superficial understanding. The theory of deep learning originates from Benjamin Samuel Bloom’s (1956) cognitive hierarchy theory, which proposes a progression of cognitive activities from “lower-order” to “higher-order” levels, emphasizing learners’ active participation in “meaning construction” rather than passive information reception. Marton and Säljö (18) introduced the concept of deep learning based on their research categorizing students’ reading approaches into deep and surface intentions and strategies. Compared to surface learning, deep learning involves a deliberate process in which learners actively interpret information to establish connections with prior knowledge, synthesize ideas into coherent frameworks, and ultimately solve complex problems, thereby enabling innovative decision-making (19, 20). Centered on the concept of deep learning, educational theories have developed multiple interrelated core perspectives that reveal the essence and pathways to achieving deep learning from dimensions such as cognitive processes, learning objectives, and instructional conditions. Extensive research has shown that adopting deep learning approaches helps learners develop higher-order cognitive skills, including critical thinking and integrative thinking (21, 22). Students with such advanced cognitive abilities are better equipped to overcome challenges associated with online learning, such as limited interaction in asynchronous environments (23). Furthermore, deep learning has been demonstrated to positively impact learning outcomes and support students’ long-term career development (24). In the field of nursing education, experts advocate for the integration of deep learning strategies, including ontological and reflective approaches, as effective means to cultivate proficient nursing practitioners (25).

In comparison to traditional face-to-face classrooms, online or hybrid learning environments offer distinct advantages in fostering interaction both between instructors and students, as well as among students themselves (26, 27). The accessibility of online communication encourages students to express themselves more freely, mitigating the inhibitions of in-person settings and effectively addressing the common deficit of teacher-student interaction in higher education (28). Effective instructional interaction is a critical mechanism for fostering deep learning (29). By strengthening multifaceted exchanges, it not only enhances students’ motivation for and engagement in deep learning but also consolidates their application of deep learning strategies (30), thereby improving overall learning outcomes. Existing research has demonstrated that classroom interaction plays a key role in stimulating deep learning and the development of higher-order thinking skills (31). Collaborative tasks, in particular, are more likely to stimulate interaction among students compared to individual assignments (32), and group discussions in collaborative learning settings are particularly conducive to deep learning (33). Furthermore, deep learning has been shown to mediate the relationship between teaching strategies and learning outcomes. However, much of the existing literature has focused on the direct effects of teaching methods on deep learning, leaving the mediating role of deep learning in the context of online real-time teaching interactions and teaching effect largely unexplored. This study seeks to investigate the mediating role of deep learning between online real-time teaching interaction and teaching effect among undergraduate nursing students, offering practical insights for the design of synchronous online courses and valuable insights into the advancement of nursing education.

The research hypotheses for this study are as follows:

Hypothesis 1: There is a positive correlation between online real-time teaching interaction and teaching effect among undergraduate nursing students.

Hypothesis 2: Deep learning is positively associated with teaching effect among undergraduate nursing students.

Hypothesis 3: There is a positive relationship between the online real-time teaching interaction and deep learning among undergraduate nursing students.

Hypothesis 4: Deep learning mediates the relationship between online real-time teaching interaction and teaching effect among undergraduate nursing students.

## 2 Objects and methods

### 2.1 Participants

Participants were selected from the universities in Hebei Province offering nursing programs, utilizing a convenience sampling method. Inclusion criteria encompassed full-time undergraduate nursing students engaged in online learning, with normal logical thinking abilities, who provided informed consent and volunteered to participate. Exclusion criteria included undergraduate nursing students who had either left the university or taken leave due to illness or other reasons. The study received approval from the Ethics Committee of North China University of Science and Technology (approval number 2025090). Data were collected via WJX, a professional online survey platform widely used in China, with over 90% of higher education institutions utilizing the platform. Before participation, all participants were informed of the study’s objectives, and measures were implemented to ensure the authenticity and anonymity of the responses. Consequently, identifying information was not accessible. Upon providing consent, participants were granted access to the survey by scanning a designated QR code, enabling them to complete the questionnaire by selecting their responses. In this study, the sample size was calculated based on the finite population cross-sectional formula $n=\frac{{\left(\frac{Z_{\alpha }⋆\sigma }{\delta }\right)}^{2}}{1+{\left(\frac{Z_{\alpha }⋆\sigma }{\delta }\right)}^{2}/N}\;$ , and considering a 5% rate of invalid questionnaires, it was determined that the sample size should be at least 499 participants. 623 questionnaires were collected over a one-week period (from September 11, 2025 to September 18, 2025). Considering that the questionnaire omissions were completely random and the total number of omissions was not large 36, the direct list deletion method was adopted to handle cases containing any missing values. The final effective sample size used for the SEM analysis was 587 (The effective response rate was 94.2%), which was still much higher than the sample size requirement estimated by the model.

### 2.2 Measures

#### 2.2.1 General information questionnaire

Building upon relevant literature (34, 35), the researchers developed a general information questionnaire that included eight variables: gender, academic year, place of household registration, whether the participant is an only child, involvement in student leadership roles, primary choice of nursing as a major, area of specialization, and the online learning platforms most frequently used. The “Online Real-time Teaching Interaction Scale,” “Deep Learning Scale” and “Teaching Effect Scale” adopted in this study are all mature scales that have been developed and widely used in the context of nursing education research in China (as illustrated in Supplementary Table S1). The selection of these scales is based on their solid theoretical foundation and the psychometric attributes that have been verified in previous studies. To ensure its applicability and reliability in the sample of this study, we carried out the following verification steps: (1) Content validity confirmation: Three nursing education experts were invited to evaluate the applicability of the scale items in the online teaching context of this study. All items were considered relevant in content and clearly expressed. (2) Revalidation of reliability and structural validity: Analyze based on the formal sample data of this study. Reliability analysis revealed that the Cronbach’s alpha coefficients for each scale exceeded 0.92, demonstrating a high level of internal consistency. The results of confirmatory factor analysis support the original factor structure of each scale, and all model fitting indicators meet the acceptable standards (for example, CFI > 0.90, TLI > 0.90, RMSEA < 0.1), confirming its good structural validity.

#### 2.2.2 Online real-time teaching interaction scale

The online real-time teaching interaction scale was adapted by Cheng et al. (36). The scale comprises 31 items employing a 5-point Likert-type response format, ranging from 1 (extremely inconsistent) to 5 (extremely consistent), with a total score range of 31 to 155 points. It evaluates online real-time teaching interaction across four dimensions: human-media interaction, teacher-student interaction, peer-to-peer interaction, and self-interaction. The internal consistency of the scale was assessed through Cronbach’s alpha, yielding a coefficient of 0.954, indicating a high level of reliability in this study.

#### 2.2.3 Deep learning scale

The deep learning scale developed by SDL (37) and subsequently revised by Cheng et al. (36) was employed to assess the deep learning attributes of nursing students in this study. The scale evaluates deep learning across three dimensions: personal cognition, self-regulation, and interpersonal coordination. It consists of 12 items, including statements such as “Through online synchronous learning, I have acquired solid professional knowledge and skills” and “Through online synchronous learning, my ability to solve complex problems has improved.” A five-point Likert scale was used, with responses ranging from 1 (“extremely inconsistent”) to 5 (“extremely consistent”). The total score ranges from 12 to 60, with higher scores indicative of greater levels of deep learning. The internal consistency of the deep learning scale in this study was demonstrated by a Cronbach’s alpha coefficient of 0.922.

#### 2.2.4 Teaching effect scale

The teaching effect scale, initially developed by Li et al. (38) and later utilized by Wu et al. (35), was employed to assess the teaching effect of nursing students in this study. The scale consists of 22 items, organized into four dimensions: learning engagement, learning activities and cooperative exchanges, learning outcomes, and classroom evaluation and recognition. Participants rated each item using a 5-point Likert-type scale, ranging from 1 to 5, with a sample item being: “Online real-time teaching interaction offers a variety of learning activities (such as real-time discussions, group collaboration, case reflection, etc.).” The total score ranges from 22 to 130, with higher scores reflecting enhanced teaching effect. In this study, the Cronbach’s alpha for the overall scale was 0.958, indicating excellent internal consistency.

### 2.3 Statistical methods

The statistical analysis for this study was performed using SPSSAU. Descriptive data are presented as frequencies with percentages for categorical variables and means ± standard deviations for continuous, normally distributed variables. Pearson correlation analysis was employed to examine the relationships among undergraduate nursing students’ teaching effect, online real-time teaching interaction, and deep learning. Multiple linear regression was employed to assess the impact of online real-time teaching interaction and deep learning on teaching effect. Furthermore, structural equation modeling (SEM) was employed, utilizing the maximum likelihood estimation method. The significance of the proposed mediating effects was assessed using the Bootstrap method, with a significance threshold set at *α* = 0.05. To ensure the validity of the data analysis, the skewness and kurtosis coefficients of each variable were calculated. According to statistical conventions, when the absolute value of skewness is less than 3 and the absolute value of kurtosis is less than 10, the data can be regarded as approximately normally distributed. In this study, the skewness and kurtosis of all variables are within this safe range, as illustrated in Supplementary Table S2. To evaluate whether there is a serious multicollinearity problem among the independent variables in the regression model, the variance inflation factor (VIF) and Tolerance (Tolerance) were calculated. The results show that the mean value of VIF for all independent variables is 1.113, and the corresponding tolerance values are all 0.899, indicating that there is no serious multicollinearity problem. This indicates that the linear relationship between the independent variables is relatively weak and will not have a significant impact on the estimation of model parameters. To assess the influence of demographic factors, the correlations (or inter-group comparisons) between variables such as gender and grade and online real-time teaching interaction, deep learning, and teaching effectiveness were examined. The results showed that gender and age had no significant correlation with the above core variables (Supplementary Table S3). This indicates that in the sample of this study, demographic characteristics are not systematic influencing factors of the core research variables. In the model, online real-time teaching interaction, deep learning, and teaching effectiveness are all treated as latent variables and measured by their corresponding scale items (observation indicators).

## 3 Results

### 3.1 General demographic data of nurses students

The study included 587 undergraduate nursing students: 122 males (20.78%) and 465 females (79.22%). The distribution of academic year consisted of 137 freshmen (23.34%), 155 sophomores (26.41%), 127 juniors (21.64%), and 168 seniors (28.62%). There were 277 people (47.19%) from urban households and 310 people (52.81%) from rural households. There were 144 only children (24.53%) and 443 non-only children (75.47%). There were 114 student cadres (19.42%) and 473 non-student cadres (80.58%). The first vow is to serve 224 students as guardians (38.16%), 363 students (61.84%) did not choose nursing as their first choice.

### 3.2 Common method bias test

To assess the potential for common method bias in the current study, two analytical approaches were employed. Initially, exploratory factor analysis was conducted on the questionnaire items using Harman’s single-factor test. The analysis revealed 10 factors with eigenvalues exceeding 1, collectively explaining 66.530% of the total variance. Notably, the first factor accounted for 29.355% of the variance, which is below the commonly accepted threshold of 40%. Subsequently, confirmatory factor analysis was performed by loading all items onto a single factor; however, the model demonstrated inadequate fit, with *χ*^2^ = 16202.785, df = 2015, CFI = 0.435, TLI = 0.45, RMSEA = 0.110, SRMR = 0.139. These findings suggest that the common method bias is not a significant concern in this study, allowing for further analyses to proceed (39, 40).

### 3.3 Online real-time teaching interaction, deep learning and teaching effect scores among nurse students

This study involved 587 undergraduate nursing students, with the following mean scores: online real-time teaching interaction (3.613 ± 0.751), teaching effect (3.508 ± 0.805), and deep learning (3.480 ± 0.888), as detailed in Table 1.

**Table 1:** Scores of online real-time teaching interaction, deep learning and teaching effect of undergraduate nursing students (*n* = 587).

Dimensionality	Total score ± standard deviations	Item mean score ± standard deviations
Online real-time teaching interaction	112.242 ± 23.410	3.613 ± 0.751
Human-media interaction	17.738 ± 4.757	3.548 ± 0.951
Teacher-student interaction	29.010 ± 6.873	3.626 ± 0.859
Interaction among students	33.009 ± 8.336	3.668 ± 0.926
Self-interaction	32.486 ± 8.911	3.610 ± 0.990
Deep learning	41.760 ± 10.656	3.480 ± 0.888
Personal cognition	13.990 ± 4.138	3.497 ± 1.034
Self-regulation	13.964 ± 3.976	3.491 ± 0.994
Interpersonal coordination	13.806 ± 4.136	3.451 ± 1.034
Teaching effect	77.405 ± 19.759	3.508 ± 0.805
learning engagement	14.221 ± 4.187	3.555 ± 1.047
Learning activities and cooperative exchanges	21.302 ± 6.095	3.550 ± 1.016
Learning effect	28.116 ± 7.900	3.514 ± 0.987
Classroom evaluation and recognition	13.767 ± 4.346	3.442 ± 1.086

### 3.4 Analysis of the relationship between online real-time teaching interaction, teaching effect, and deep learning among undergraduate nursing students

There was a significant positive correlation between teaching effect and online real-time teaching interaction among undergraduate nursing students (*r* = 0.398, *p* < 0.01). Similarly, a significant positive correlation existed between deep learning and teaching effect among these undergraduate nursing students (*r* = 0.407, *p* < 0.01), as illustrated in Table 2.

**Table 2:** Pearson correlation coefficients among teaching effect, online real-time teaching interaction, and deep learning (*n* = 587).

Dimensionality	Teaching effect	Learning engagement	Learning activities and cooperative exchanges	Learning effect	Classroom evaluation and recognition
Online real-time teaching interaction	0.398**	0.355**	0.351**	0.360**	0.319**
Human-media interaction	0.310**	0.255**	0.274**	0.279**	0.272**
Teacher-student interaction	0.278**	0.271**	0.232**	0.259**	0.209**
Interaction among students	0.260**	0.226**	0.236**	0.229**	0.217**
Self-interaction	0.422**	0.377**	0.376**	0.384**	0.329**
Deep learning	0.407**	0.350**	0.358**	0.413**	0.258**
Personal cognition	0.354**	0.304**	0.298**	0.364**	0.238**
Self-regulation	0.352**	0.294**	0.308**	0.366**	0.221**
Interpersonal coordination	0.355**	0.314**	0.328**	0.349**	0.215**

**p < 0.01.

### 3.5 Multivariate linear regression analysis of the teaching effect among undergraduate nursing students in this department

The teaching effect score was treated as the dependent variable, with online real-time teaching interaction and deep learning scores as independent variables in the multiple linear regression analysis. The findings indicated that the combined influence of online real-time teaching interaction and deep learning accounted for 18.5% of the total variance in the teaching effect among undergraduate nursing students, as shown in Table 3. The learning outcomes of nursing students represent a multifaceted construct shaped by a variety of interrelated factors. In addition to the dynamics of teaching interactions and cognitive processes, key influences include individual motivation, prior knowledge, the campus environment, clinical practice experiences, and non-cognitive skills. This study specifically examines the interplay between two pivotal variables: online real-time teaching interaction and deep learning. It demonstrates that these factors collectively account for nearly one-fifth of the variance in teaching effectiveness. The findings elucidate a crucial operational mechanism: educators can effectively promote students to enter a deeper learning process by systematically designing and optimizing online real-time interaction strategies, thereby enhancing their teaching effectiveness as a whole. This discovery provides nursing educators with clear, specific and evidence-based directions for teaching improvement.

**Table 3:** Linear regression analysis predicting teaching effect with online real-time interaction and deep learning (*n* = 587).

Predictors	β	*b*	*SE*	*t*	95% CI
Constant	—	2.108	0.134	15.680**	1.844 ~ 2.372
Online real-time teaching interaction	0.363	0.339	0.043	7.968**	0.255 ~ 0.422
Deep learning	0.107	0.091	0.039	2.357*	0.015 ~ 0.166
Δ*R*^2^	0.185				
*F*	F (2,620) = 67.460, *p* = 0.000				

*p < 0.05; **p < 0.01.

### 3.6 Analysis of the mediating effect of deep learning between online real-time teaching interaction and the teaching effect of undergraduate nursing students

**Figure 1:**
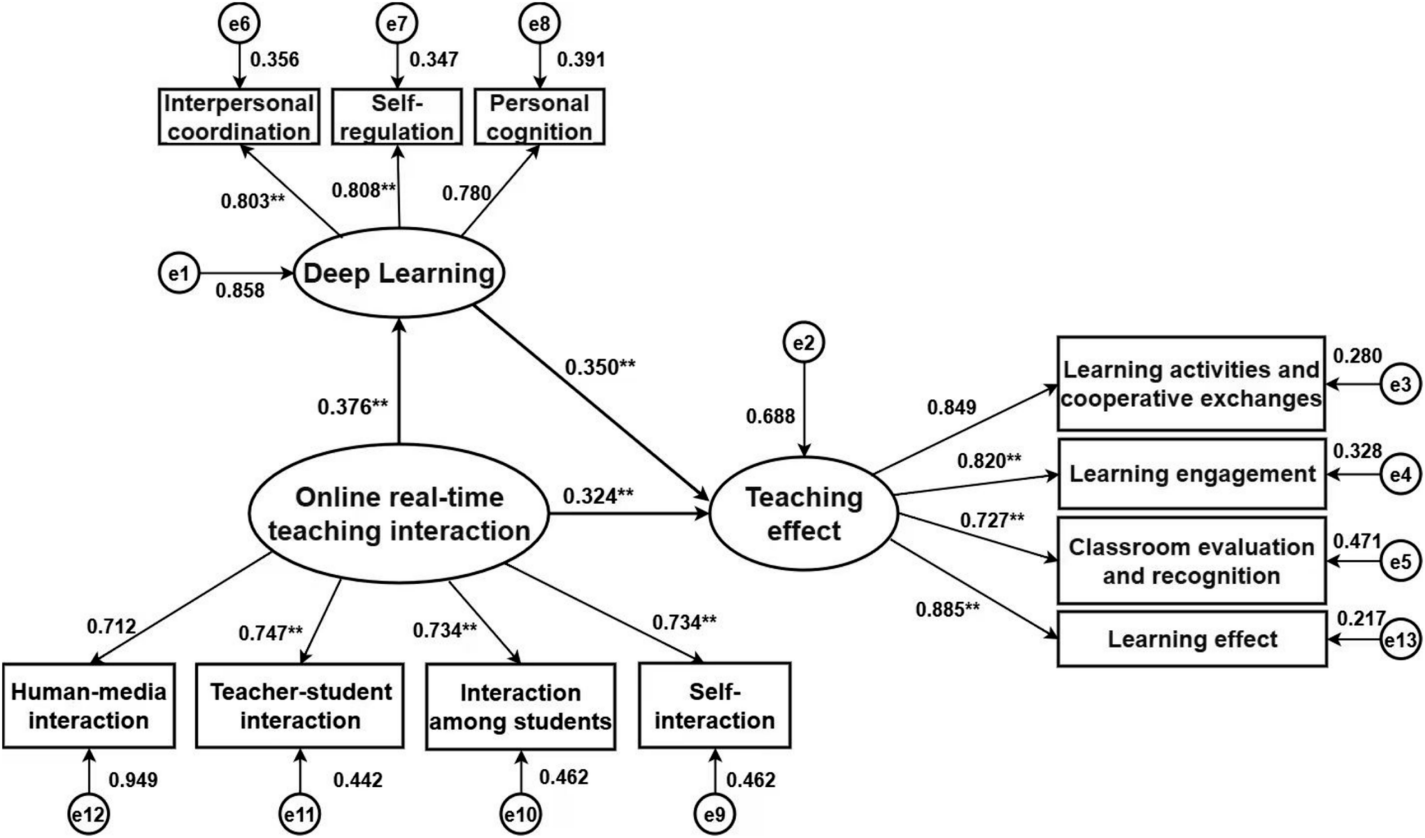
Structural equation model (standardized) of the relationship between deep learning, online real-time teaching, and teaching effect among undergraduate nursing students.

Building upon the correlation analysis results, a hypothesized model was developed, positioning teaching effect as the dependent variable, online real-time teaching interaction as the independent variable, and deep learning as the mediating variable (refer to Figure 1). All fit indices adhered to established thresholds, confirming a robust model fit, as detailed in Table 4. The Bootstrap method was employed to assess the significance of deep learning’s mediating role. The results revealed that the 95% confidence interval for the mediating effect of deep learning between online real-time teaching interaction and teaching effect ranged from 0.108 to 0.200, excluding zero, thereby confirming the significance of this mediation. The direct effect of online real-time teaching interaction on teaching effect among undergraduate nursing students was 0.289, while the indirect effect through deep learning was 0.165, accounting for 36.344% of the total effect, as presented in Table 5.

**Table 4:** Model fit indices and their acceptable thresholds for structural equation modeling (SEM).

Index of fit	TLI	AGFI	IFI	The chi-square degrees of freedom ratio χ^2^/*df*	GFI	RMSEA	RMR
Judging standard	>0.9	>0.9	>0.9	<3	>0.9	<0.10	<0.05
Value	0.984	0.961	0.988	1.928	0.976	0.040	0.041
Index of fit	PGFI	PNFI	PCFI	SRMR	CFI	NFI	NNFI
Judging standard	>0.5	>0.5	>0.5	<0.1	>0.9	>0.9	>0.9
Value	0.606	0.727	0.736	0.041	0.988	0.975	0.984

**Table 5:** Bootstrap analysis of the mediation effect of deep learning between online real-time teaching interaction and teaching effect (*n* = 587).

Parameters	Estimates of value	Lower limit	Upper limit	*p*	Effect proportion/%
Indirect effect	0.165	0.108	0.200	0.000	36.344
Direct effect	0.289	0.218	0.361	0.000	63.656
Total effect	0.454	0.375	0.533	0.000	100.000

## 4 Discussion

### 4.1 The situation teaching effect for undergraduate nursing students

The results of this study reveal a generally favorable perception of teaching effect among undergraduate nursing students, with a total score of 77.405 ± 19.759 and a mean item score of 3.508 ± 0.805. This mean score, which exceeds two-thirds of the maximum possible score per item, aligns with previous studies (35). For example, a meta-analysis on blended learning showed that it outperforms traditional teaching in terms of knowledge acquisition (SMD = 0.64), skill performance (SMD = 0.37), and learning satisfaction (SMD = 0.32) (41). Another meta-analysis on the BOPPPS teaching model also indicated that this model significantly improves students’ theoretical scores (MD = 3.35) and practical scores (MD = 4.50), with student satisfaction reaching as high as 89% (42). A further dimensional analysis revealed that learning engagement scored the highest (3.555 ± 1.047), followed by learning activities & collaborative communication and learning Outcomes. Conversely, the lowest score was recorded for Classroom Evaluation and Recognition (3.442 ± 1.086), a difference that may be attributed to the environmental limitations inherent in online learning, such as variations in personal hardware and network stability. A closer examination of the components of online real-time interaction revealed that human-media interaction yielded the lowest score (3.548 ± 0.951). This finding is significant, as effective human-media interaction—which involves an intuitive and functional platform interface as well as well-structured, accessible instructional content—is a key determinant of teaching effect (43). Rather than simply delivering content, it shapes students’ cognitive engagement with the material. Especially in the teaching of nursing skills, the real-time observation and correction of operational details in the online environment have inherent limitations, which may be the professional situational factor leading to the relatively low evaluation of this dimension. Therefore, the availability of teaching platforms and the design quality of digital resources directly affect students’ efficiency in processing and integrating knowledge (44). This also suggests that insufficient human-media interaction may become a key bottleneck in current online teaching, restricting the improvement of the overall teaching effect (45). In the future, in blended teaching, it is necessary to enhance offline or high-fidelity simulation links to make up for the deficiencies of online assessment.

### 4.2 Online real-time teaching interaction can positively influence the online teaching effect among undergraduate nursing students

This study indicates that the overall score of nursing undergraduates in online real-time teaching interactions was at a medium-high level (total score: 112.242 ± 23.410, mean score: 3.613 ± 0.751), suggesting a generally favorable quality of interaction. This result is similar to the research findings of Hong et al. (46) which pointed out that undergraduate nursing students have a relatively high demand for interaction in online courses, especially valuing discussions between teachers and students (81.75%). Further analysis revealed that student–student and teacher-student interaction scores were particularly prominent, reflecting that in synchronous online teaching environments, teacher-student and student–student interactions remain core elements of instructional implementation. This reflects that in synchronous online teaching environments, the core elements of instructional implementation align with the Community of Inquiry framework, which posits that teaching presence and social presence are foundational for meaningful learning (47). This finding further validates the importance of teacher-student interaction in online teaching (48) and extends this perspective by emphasizing the unique role of peer interaction, which is crucial for collaborative knowledge construction (49). Statistical results showed a significant positive correlation between online real-time teaching interactions and teaching effect (*p* < 0.01), indicating that increasing online real-time interactions can significantly enhance the learning outcomes of nursing undergraduates and has clear positive predictive value. This finding not only aligns with the research of Chen et al. (50) but also reinforces the critical role of interaction in improving teaching effect.

In existing online teaching research, although teacher-student interaction has been extensively studied (51), fewer studies have focused on the contributions of human-media interaction, peer interaction, and self-interaction to teaching effect. The results of this study reveal the impact of multiple dimensions of interaction in online teaching on teaching effect, particularly highlighting the key roles of self-interaction, human-media interaction, and peer interaction. Self-interaction, which refers to learners’ self-reflection, knowledge construction, and self-evaluation during the learning process, plays a significant role in deep understanding and internalization of knowledge (52). The high scores in this dimension indicate that nursing undergraduates can promote their own learning progress through autonomous learning and self-feedback in online teaching, thereby enhancing learning outcomes (53). Human-media interaction involves learners’ interaction with technological platforms, such as submitting tasks, searching for materials, and taking online tests through learning management systems or teaching platforms. Research shows that with technological advancements, the design and functionality of learning platforms increasingly influence learning outcomes (54). In this study, high-quality human-media interaction showed a strong positive correlation with teaching effect, implying that optimizing the interactive functions of online teaching platforms and providing personalized learning support can effectively enhance undergraduate nursing students’ learning motivation and outcomes. Peer interaction offers learners opportunities for communication and collaboration, which not only facilitates co-construction of knowledge but also increases classroom engagement and a sense of community (55). In online nursing education, peer feedback and collaboration significantly strengthen students’ mastery of subject matter and support deeper discussion and reflection when addressing complex problems.

### 4.3 The mediating role of deep learning between online real-time teaching interaction and teaching effect among undergraduate nursing students

In this study, the average score of deep learning for undergraduate nursing students was 3.480 ± 0.888, which was also at an above-average level. This level is highly consistent with the research results of Wang et al. on Chinese undergraduate nursing students, indicating that undergraduate nursing students in China generally have a good tendency towards deep learning (56). The results of this study indicate that deep learning plays a significant mediating role between online real-time teaching interaction and teaching effectiveness among undergraduate nursing students, highlighting its importance in enhancing teaching outcomes. This finding aligns with existing literature, indicating that online real-time teaching interaction facilitates active learning and improves deep learning capabilities among nursing undergraduates, thereby promoting the development of professional competencies (57). The mediating effect of deep learning is significant across three dimensions: individual cognition, self-regulation, and interpersonal coordination. Through online real-time interaction, students’ initiative and knowledge transfer abilities are stimulated at the individual cognitive level, significantly enhancing their deep learning capacity and subsequently fostering the development of professional practical skills (58). The approach of online real-time interaction helps learners improve their ability to plan, monitor, and reflect on their own learning processes, thereby significantly enhancing their professional learning capabilities. Deep learning is not an isolated process but rather achieves knowledge co-construction and perspective expansion through social interaction, serving as a contextual safeguard for deep learning. Compared to traditional passive learning models, online real-time teaching interaction provides a more flexible and interactive learning platform. On this platform, students can engage in instant communication and feedback with instructors and peers, participate in diverse learning activities, and thereby construct a richer learning environment (59). This interactivity significantly enhances students’ learning interest and spirit of inquiry, further promoting the internalization and transfer of knowledge (60).

Deep learning is particularly important in practice-intensive disciplines such as nursing. Nursing education requires students not only to master substantial theoretical knowledge but also to apply this knowledge in real clinical situations to solve practical problems. Therefore, undergraduate nursing students’ deep learning is directly related to the cultivation of their professional skills. Supported by online real-time teaching interactions, undergraduate nursing students’ deep learning capacity is significantly enhanced, which not only optimizes their academic performance but also facilitates the practical application of their professional skills. Existing literature has shown that deep learning helps students better cope with complex situations and solve problems in practice (61). This study further verifies the positive role of deep learning in improving nursing students’ clinical competencies. The enhancement of deep learning is closely related not only to academic performance but also directly affects students’ learning satisfaction and their perception of teaching effect. Nursing students often face high levels of academic pressure and practical challenges. Improving their deep learning ability can help them better cope with these challenges and enhance their satisfaction with online teaching (62). This is consistent with conclusions in existing literature stating that students’ learning experiences and their evaluation of teaching quality depend, to some extent, on their deep learning capabilities.

Therefore, nursing educators should actively adopt online real-time teaching interactions and fully leverage their advantages, such as flexibility, interactivity, and immediate feedback. By designing highly interactive teaching activities, instructors can not only increase student engagement but also monitor learning progress in real time and adjust teaching strategies based on student feedback. This real-time flexibility provides personalized learning support, fosters the development of deep learning abilities, and plays a crucial role in deepening students’ professional knowledge and enhancing their practical competencies.

## 5 Countermeasures and suggestions

Firstly, the course development team should carefully design interactive tasks oriented towards questions or cases to ensure they are closely aligned with the goals of deep learning and stimulate the interest of undergraduate nursing students in exploration. During the task design process, the course development team should also take into account the learning needs of different nursing students, rationally allocate the difficulty of tasks, avoid setting tasks that are too single or too complex, ensure that students can maintain a positive learning attitude during the task execution process, and gradually improve their level of deep learning. Secondly, the technical support department needs to optimize the functions of the online teaching platform, and utilize technical means such as group discussion rooms, real-time feedback tools, and learning analysis dashboards to provide support for efficient and traceable teaching interactions. Finally, a regular feedback and iteration mechanism should be established throughout the teaching process. The actual effect of teaching activities is evaluated from multiple dimensions and perspectives through the collection of learning and analysis data from the system, student satisfaction questionnaires, and qualitative evaluations by teaching supervisors.

## 6 Limitations

Firstly, although the study controlled for some basic confounding factors, such as the grade of the undergraduate nursing students, whether they held student leadership positions, and whether their first choice, it failed to cover all potential variables, such as learning style and prerequisite course grades. Future research should incorporate more relevant covariates to more accurately estimate the net effect of online real-time teaching interaction on teaching effect among undergraduate nursing students. Secondly, the samples of this study were only sourced from a few universities in the Hebei province. Due to the limitations of region, type of institution and teaching resources, the universality of the research conclusion may be affected. Future research can take samples from a wider geographical area and institutions of different levels to test the representativeness of the results. Finally, this study mainly uses scales to measure the effectiveness of deep learning and teaching. Although it ensures the validity of the measurement, this method is difficult to fully capture the complex and dynamic psychological and behavioral changes in the process of teaching interaction and deep learning. Future research can combine qualitative methods such as interviews and classroom observations to conduct a more in-depth and comprehensive assessment of the learning psychology and behavioral mechanisms of undergraduate nursing students.

## Data Availability

The original contributions presented in the study are included in the article/Supplementary material, further inquiries can be directed to the corresponding author.
