# Supplementary material for: The impact of online real-time teaching interaction on teaching effect among undergraduate nursing students: the mediating role of deep learning
Source: Front Public Health. 2026 Jan 30;14:1715936. doi: 10.3389/fpubh.2026.1715936 (PMC12903777; doi:10.3389/fpubh.2026.1715936)
Supplement: Supplementary file 1 [file Table_1.docx]

Supplementary Material

# Supplementary Tables

## **Table S1**

## Questionnaire on the impact of online real-time teaching interaction on the teaching effect of undergraduate nursing students

| ****Part One:** Basic information** | | | | | |
| --- | --- | --- | --- | --- | --- |
| 1. Your gender | \| ○ Male \| ○ Female \| \| --- \| --- \| | | | | |
| 2. Your academic year | \| ○ Freshman  ○ Sophomore \| ○ Junior  ○ Senior \| \| --- \| --- \| | | | | |
| 3. Registered permanent residence (Hukou location) | \| ○ Urban household registration  ○ Rural household registration \| \| --- \| | | | | |
| 4. Are you an only child? | \| ○Yes ○No \| \| --- \| | | | | |
| 5. Are you a student leader? | ○Yes ○No | | | | |
| 6. Was nursing your first choice of major? | \| ○Yes ○No \| \| --- \| | | | | |
| 7. Your major/specialization (you may select multiple options) | \| ○Adult Nursing  ○Pediatric Nursing  ○Obstetric and Gynecological Nursing \| ○Psychiatric Nursing  ○Community Nursing  ○Other _____ \| \| --- \| --- \| | | | | |
| 8. Online learning platforms you frequently use (you may select multiple options) | \| ○Tencent Meeting (VooV Meeting)  ○Rain Classroom (Yuketang)  ○Superstar Learning Platform (Chaoxing)  ○DingTalk \| ○Instant Messaging Tools (e.g., WeChat/QQ)  ○Virtual Simulation Platform (e.g., Nursing Skill Simulation System)  ○Other _________ \| \| --- \| --- \| | | | | |
| **Part Two: Online Real-time Teaching Interaction**  (The following questions are all about the teaching interaction you experienced when participating in online learning. Please truthfully select according to your own feelings.) | | | | | |
| **9. Human–Media Interaction (Interaction with Platform/Resources)** | | | | | |
|  | extremely inconsistent | Inconsistent | Neutral | Consistent | extremely consistent |
| 9.1. In online synchronous learning, the learning platform’s functions are comprehensive and can basically meet my learning needs. | ○ | ○ | ○ | ○ | ○ |
| 9.2. In online synchronous learning, I can switch between platform pages smoothly and become proficient in using its features. | ○ | ○ | ○ | ○ | ○ |
| 9.3. In online synchronous learning, I can understand the fundamental principles of the learning content and the relationships among different components. | ○ | ○ | ○ | ○ | ○ |
| 9.4. In online synchronous learning, I can engage in reflective learning guided by the learning resources. | ○ | ○ | ○ | ○ | ○ |
| 9.5. In online synchronous learning, the learning resources cover multiple dimensions, significantly enhancing my ability to extend knowledge and think creatively. | ○ | ○ | ○ | ○ | ○ |
| **10.** Teacher–Student Interaction | | | | | |
| 10.1 In online synchronous learning, the teacher effectively communicates course information (e.g., class schedule, course content). | ○ | ○ | ○ | ○ | ○ |
| 10.2. In online synchronous learning, the teacher uses multiple methods to clearly convey concepts. The teacher also helps me resolve technical platform issues. | ○ | ○ | ○ | ○ | ○ |
| 10.3. In online synchronous learning, the teacher provides timely assistance in answering course-related questions. | ○ | ○ | ○ | ○ | ○ |
| 10.4. In online synchronous learning, the teacher organizes course content logically, with appropriate distribution of course load and knowledge points. | ○ | ○ | ○ | ○ | ○ |
| 10.5. In online synchronous learning, the teacher pays attention to my participation in the chat zone or other communication tools and responds accordingly. | ○ | ○ | ○ | ○ | ○ |
| 10.6. In online synchronous learning, the teacher allows me to express ideas and engage in brainstorming during online classes or through communication tools. | ○ | ○ | ○ | ○ | ○ |
| 10.7. In online synchronous learning, the online learning resources provided by the teacher meet my learning needs. | ○ | ○ | ○ | ○ | ○ |
| 10.8. In online synchronous learning, the teacher effectively motivates my learning engagement. | ○ | ○ | ○ | ○ | ○ |
| **11.** Student–Student Interaction | | | | | |
| 11.1. In online synchronous learning, I can ask questions about unclear content and receive timely feedback. | ○ | ○ | ○ | ○ | ○ |
| 11.2. In online synchronous learning, I obtain additional shared resources from other learners in this course. | ○ | ○ | ○ | ○ | ○ |
| 11.3. In online synchronous learning, I can communicate with other learners about course content through various means such as chat messages or verbal comments. | ○ | ○ | ○ | ○ | ○ |
| 11.4. In online synchronous learning, I can engage in focused discussions with peers on specific topics. | ○ | ○ | ○ | ○ | ○ |
| 11.5 In online synchronous learning, I share my thoughts and applications of the course content with other students. | ○ | ○ | ○ | ○ | ○ |
| 11.6. In online synchronous learning, I receive motivational information regarding reflection and summary from my peers. | ○ | ○ | ○ | ○ | ○ |
| 11.7. The group activities in online synchronous classes provide me with opportunities to interact with classmates. | ○ | ○ | ○ | ○ | ○ |
| 11.8. In online synchronous learning, I can use multiple tools to offer constructive and feasible suggestions on peer assignments and improvement strategies. | ○ | ○ | ○ | ○ | ○ |
| 11.9. In online synchronous learning, interacting with other students effectively helps resolve my learning difficulties and enhances my learning ability. | ○ | ○ | ○ | ○ | ○ |
| 12. ****Self-Interaction**** | | | | | |
| 12. 1. In online synchronous learning, I frequently take notes on important concepts, content, or viewpoints. | ○ | ○ | ○ | ○ | ○ |
| 12. 2. In online synchronous learning, I gather information from multiple sources to deepen my understanding of the course content. | ○ | ○ | ○ | ○ | ○ |
| 12. 3. In online synchronous learning, I attempt to compare, connect, and integrate my own ideas with the course material. | ○ | ○ | ○ | ○ | ○ |
| 12. 4. In online synchronous learning, I promptly upload summaries and reflections of my learning. | ○ | ○ | ○ | ○ | ○ |
| 12. 5. In online synchronous learning, I constantly monitor whether I am adhering to my study plan and adjust or improve my learning behaviors accordingly. | ○ | ○ | ○ | ○ | ○ |
| 12. 6. In online synchronous learning, I frequently reflect on and evaluate my learning through timely summarize. | ○ | ○ | ○ | ○ | ○ |
| 12. 7 In online synchronous learning, when encountering setbacks, I promptly find strategies to regain confidence and maintain motivation. | ○ | ○ | ○ | ○ | ○ |
| 12. 8 In online synchronous learning, I strive to arrange a relatively comfortable environment for studying. | ○ | ○ | ○ | ○ | ○ |
| 12. 9. In online synchronous learning, I am able to fully immerse myself in the learning process. | ○ | ○ | ○ | ○ | ○ |
| ****Part three: Deep Learning**** (The following items assess your experience of deep learning during online synchronous learning. Please select based on your personal perception.) | | | | | |
| **13. Personal Cognition** | | | | | |
| 13. 1. In online synchronous learning, I have acquired solid professional knowledge and skills. | ○ | ○ | ○ | ○ | ○ |
| 13. 2. In online synchronous learning, I am able to distinguish truth from falsehood, identify the essence of problems, and make reasonable decisions. | ○ | ○ | ○ | ○ | ○ |
| 13. 3. In online synchronous learning, my ability to solve complex problems has improved. | ○ | ○ | ○ | ○ | ○ |
| 13. 4. In online synchronous learning, I have gained new insights into my major and am willing to continue exploring knowledge related to it. | ○ | ○ | ○ | ○ | ○ |
| **14. Self-Regulation** | | | | | |
| 14.1. In online synchronous learning, I have greater confidence in my learning abilities. | ○ | ○ | ○ | ○ | ○ |
| 14.2. In online synchronous learning, I am able to persistently complete my learning goals. | ○ | ○ | ○ | ○ | ○ |
| 14.3. In online synchronous learning, I can independently formulate a study plan, monitor its implementation, and adjust it promptly. | ○ | ○ | ○ | ○ | ○ |
| 14.4. In online synchronous learning, my ability to acquire, screen, and extract information has improved. | ○ | ○ | ○ | ○ | ○ |
| **15.** **Interpersonal Coordination** | | | | | |
| 15.1. In online synchronous learning, I am willing to listen to others’ opinions or feedback. | ○ | ○ | ○ | ○ | ○ |
| 15.2. In online synchronous learning, I can actively negotiate with group members to establish shared learning goals. | ○ | ○ | ○ | ○ | ○ |
| 15.3. In online synchronous learning, I am able to share my perspectives with peers. | ○ | ○ | ○ | ○ | ○ |
| 15.4. In online synchronous learning, I can express myself effectively through oral and written communication. | ○ | ○ | ○ | ○ | ○ |
| ****Part four****：**Teaching Effect Scale** | | | | | |
| **（1）Learning Engagement** | | | | | |
| 16. How many hours per week do you spend on online course learning？ | ○Less than 3 hours ○3–8 hours  ○8–15 hours ○15–20 hours  ○More than 20 hours | | | | |
| 17. What percentage of the learning materials provided on this course’s online platform (e.g., xuexitong Tencent Meeting) have you reviewed？ | ○0% ○20%  ○50% ○80%  ○100% | | | | |
| 18. Before each live online class, how frequently do you use the online teaching platform for learning? | ○Very high ○High  ○Moderate ○Low  ○Very low | | | | |
| 19. After each live online class, how frequently do you use the online teaching platform for review or extended learning? | ○Very high ○High  ○Moderate ○Low  ○Very low | | | | |
| **(2) Learning Activities and Collaborative Exchange** | | | | | |
| 20. **In the current semester’s real-time online teaching interaction model, do you agree with the following statements?** | | | | | |
| 20.1. The real-time teaching interaction provides diversified learning activities (e.g., real-time discussions, group collaboration, case reflection). | ○ | ○ | ○ | ○ | ○ |
| 20.2. Quizzes and assignments help me promptly identify and address knowledge gaps. | ○ | ○ | ○ | ○ | ○ |
| 20.3. Communicating with teachers and peers via chat, discussion boards, or messaging tools helps me resolve issues in a timely manner. | ○ | ○ | ○ | ○ | ○ |
| 20.4 Under the real-time teaching interaction model, my classroom participation and engagement have significantly increased. | ○ | ○ | ○ | ○ | ○ |
| 20.5. I actively participate in group collaborative tasks and fulfill my assigned roles. | ○ | ○ | ○ | ○ | ○ |
| 20.6. The combination of self-study and in-class discussions is well-aligned with the course content. | ○ | ○ | ○ | ○ | ○ |
| **(3) Learning Effect** | | | | | |
| **21. Compared to traditional classroom teaching, what benefits do you perceive from real-time online teaching interaction?** | | | | | |
| 21.1. Understanding and mastering foundational nursing knowledge. | ○ | ○ | ○ | ○ | ○ |
| 21.2. Expanding knowledge breadth and accessing more course resources. | ○ | ○ | ○ | ○ | ○ |
| 21.3. Enhancing learning motivation and stimulating interest. | ○ | ○ | ○ | ○ | ○ |
| 21.4. Cultivating and improving self-directed learning ability. | ○ | ○ | ○ | ○ | ○ |
| 21.5. Cultivating and improving teamwork and communication skills. | ○ | ○ | ○ | ○ | ○ |
| 21.6. Cultivating and improving clinical analysis and problem-solving abilities. | ○ | ○ | ○ | ○ | ○ |
| 21.7. Improving learning efficiency. | ○ | ○ | ○ | ○ | ○ |
| 21.8. Enhancing overall learning effectiveness. | ○ | ○ | ○ | ○ | ○ |
| **（4）Evaluation and Recognition of Real-Time Teaching Interaction** | | | | | |
| 22. Do you agree that this semester’s assessment method can comprehensively evaluate your learning performance. | ○Strongly agree ○Agree  ○Neutral ○Disagree  ○Strongly disagree | | | | |
| 23. Regarding this semester’s grading structure — Class Participation & Performance (10%) + Online Learning Engagement (15%) + Assignment Completion (15%) + Final Exam (60%) — do you find it reasonable? | ○Very reasonable ○Somewhat reasonable  ○Neutral ○Somewhat unreasonable  ○Very unreasonable | | | | |
| 24. Are you adapted to the teaching mode based on online real-time interaction? | ○Very well adapted ○Moderately adapted  ○Neutral ○Slightly unadapted  ○Very unadapted | | | | |
| 25. Would you be willing to continue using the current online real-time interactive teaching model? | ○ Very willing  ○ Somewhat willing  ○ Neutral  ○ Somewhat unwilling  ○ Very unwilling | | | | |

## **Table S2**

## Skewness and Kurtosis tests.

|  | mean score | standard deviations | Skewness | Kurtosis | Kolmogorov-Smirnov  test | | Shapiro-Wilk  test | |
| --- | --- | --- | --- | --- | --- | --- | --- | --- |
|  |  |  |  |  | Statistical value D | p | Statistical value W | p |
| teaching effect | 77.405 | 19.759 | -1.222 | -0.103 | 0.289 | 0.000** | 0.713 | 0.000** |
| deep learning | 41.760 | 10.656 | -1.171 | -0.092 | 0.307 | 0.000** | 0.764 | 0.000** |
| online real-time teaching interaction | 112.242 | 23.410 | -1.278 | 0.066 | 0.269 | 0.000** | 0.748 | 0.000** |

## **Table S3**

## Pearson correlation coefficients among gender, grade, teaching effect, online real-time teaching interaction, and deep learning (n = 587).

|  | gender | grade | teaching effect | deep learning | online real-time teaching interaction |
| --- | --- | --- | --- | --- | --- |
| gender | 1 |  |  |  |  |
| grade | 0.049 | 1 |  |  |  |
| teaching effect | -0.022 | -0.006 | 1 |  |  |
| deep learning | 0.006 | 0.017 | 0.407** | 1 |  |
| online real-time teaching interaction | 0.007 | -0.070 | 0.398** | 0.319** | 1 |

**p*<0.05 ***p*<0.01
